# WHO Trial Registration Data Set (TRDS) extension for traditional Chinese medicine 2020: recommendations, explanation, and elaboration

**DOI:** 10.1186/s12874-020-01077-w

**Published:** 2020-07-17

**Authors:** Xuan Zhang, Liang Lan, Jacky C. P. Chan, Linda L. D. Zhong, Chung-Wah Cheng, Wai-Ching Lam, Ran Tian, Chen Zhao, Tai-Xiang Wu, Hong-Cai Shang, Ai-Ping Lyu, Zhao-Xiang Bian

**Affiliations:** 1 Chinese Clinical Trial Registry (Hong Kong), Hong Kong Chinese Medicine Clinical Study Centre, The EQUATOR China Centre, School of Chinese Medicine, Hong Kong Baptist University (HKBU), Room 307, Jockey Club School of Chinese Medicine Building, 7 Baptist University Road, Kowloon Tong, Kowloon, HKSAR China; 2 Department of Computer Science, HKBU Faculty of Science, Hong Kong Baptist University, Hong Kong, SAR China; 3 Institute of Basic Research In Clinical Medicine, China Academy Of Chinese Medical Sciences, Beijing, China; 4 Chinese Cochrane Centre, West China Hospital, Sichuan University, China Trial Registration Center, Chengdu, Sichuan China; 5 Key Laboratory of Chinese Internal Medicine of Ministry of Education and Beijing, Dongzhimen Hospital, Beijing University of Chinese Medicine, Beijing, China

**Keywords:** Clinical trial registration, Chinese medicine, Recommendation, Trial registration data set (TRDS), World Health Organization (WHO)

## Abstract

**Background:**

Although the WHO Trial Registration Data Set (TRDS) has been published for many years, the quality of clinical trial registrations with traditional Chinese medicine (TCM) is still not satisfactory, especially about the inadequate reporting on TCM interventions. The development of the WHO TRDS for TCM Extension 2020 (WHO TRDS-TCM 2020) aims to address this inadequacy.

**Methods:**

A group of clinical experts, methodologists, epidemiologists, and editors has developed this WHO TRDS-TCM 2020 through a comprehensive process, including the baseline survey, draft of the initial items, three-round of Delphi survey, solicitation of comments, revision, and finalization.

**Results:**

The WHO TRDS-TCM 2020 statement extends the latest version (V.1.3.1) of TRDS published in November 2017. The checklist includes 11 extended items (including subitems), namely Source(s) of Monetary or Material Support (Item 4), Scientific Title (Item 10a and 10b), Countries of Recruitment (Item 11), Health Condition(s) or Problem(s) Studied (Item 12), Intervention(s) (Item 13a, 13b and 13c), Key Inclusion and Exclusion Criteria (Item 14), Primary and Key Secondary Outcomes (Item 19 to 20), and Lay Summary (Item B1). For Item 13 (Interventions), three common TCM interventions--i.e., Chinese herbal medicine formulas, acupuncture and moxibustion—are elaborated.

**Conclusions:**

The group hopes that the WHO TRDS-TCM 2020 can improve the reporting quality and transparency of TCM trial registrations, assist registries in assessing the registration quality of TCM trials, and help readers understand TCM trial design.

## Background

Traditional Chinese medicine (TCM), one of the oldest medical systems in the world, is widely used in China and other East Asian countries, and increasingly throughout the rest of the world [1]. TCM includes a variety of interventions, such as Chinese herbal medicines (CHM), acupuncture and moxibustion, which are variously used according to TCM’s unique theory, diagnostics and therapeutic principles [2]. Today, TCM’s impact is increasing worldwide [3]. On 25 May 2019, the World Health Organization (WHO) decided to adopt the 11th revision of the International Statistical Classification of Diseases and Related Health Problems (ICD-11). The ICD-11 adds a new category of traditional medicine conditions that involve TCM [4]. Currently, a considerable number of TCM clinical trials have been published, and most of them had been registered [5]. The purposes of clinical trial registration (CTR) are 1) to prevent publication bias; 2) to prevent unnecessary duplication of research effort, 3) to help patients and the public know what trials are planned or ongoing, and 4) to be a source of transparent data for healthcare professionals and researchers [6]. Since the first clinical trial of TCM was registered in ClinicalTrials.gov on 2 November 1999 [7], the number of TCM trial registrations has increased rapidly, especially after the requirement of mandatory trial registration proposed by the International Committee of Medical Journal Editors on 1 July 2005 [8, 9]. To standard the reporting of CTR, the WHO Trial Registration Data Set (TRDS) was issued in May 2007, specifying a minimum of 20 registration items as the international standards [10]. TRDS has been periodically updated. The latest version (V.1.3.1) has been updated to 24 items, which was issued in November 2017 [11]. In November 2018, the TRDS was further explained as “International Standards for Clinical Trial Registries” and published, with explanations of 24 items and 3 optional items [12].

Although these standard registration items have been published, the quality of reporting of TCM trial registrations is still not optimal. We have conducted a comprehensive baseline survey of TCM trial registrations from 17 WHO registries and identified 3339 eligible records from 1999 to 2017. We assessed their registration quality based on the standard items of TRDS and additional items of TCM specifics. The results revealed the poor reporting quality of TCM-related information, especially with regard to the descriptions of TCM interventions [13]. Inadequate reporting affects the readers’ judgments about the study design and undermines the purpose of trial registration [14]. In order to improve the quality of TCM clinical trials, the CONSORT (Consolidated Standards of Reporting Trials) extensions for TCM interventions, such as acupuncture [15], moxibustion [16], and CHM formulas [17], have been continually developed. Recently, the SPIRIT (Standard Protocol Items: Recommendations for Interventional Trials) for TCM extension has been published to provide suggestions in good reporting of TCM trial protocols [18]. It could be necessary to develop a reporting checklist for TCM trial registrations, as an extension of the WHO TRDS, which will beneficial to improve the quality of TCM trial registrations. Before starting the development, our working group members (ZB and TW) have introduced the rationale for establishing the guideline of TCM trial registration in workshops and academic conferences to solicit comments, as well as relevant articles have also been published [19, 20]. In addition, we consulted with professionals from the WHO International Clinical Trials Registry Platform (ICTRP) and the Chinese Clinical Trial Registry and promoted the significance of developing this extension.

## Methods

### Development of WHO TRDS-TCM 2020

As the scope of this WHO TRDS-TCM 2020 statement belongs to the reporting guideline, we referred a consensus-driven methodological framework recommended by “Guidance for developers of health research reporting guidelines” [21], but modified some steps in the development of this registration data set. Each expert has received an invitation letter by a personal email, which including the explanations of this study and informed consent for comments collection. The final participants should provide consent by accepting our email-based invitation before the survey [22]. We adopted the latest version of TRDS (V.1.3.1) as a starting point and a three-step approach is as follows:

1. ***Generating extension items through consensus meeting:***

According to our baseline survey, an TCM extension of TRDS was suggested. In reference to the explanatory documents of the previous 20-item [23] and updated 24-item of WHO TRDS [11], our working group members drafted the extension items for TCM. Then, we held a face-to-face consensus meeting with Chinese experts in Lanzhou, China, on 19 July 2017, to revise and form a checklist of reporting items.

A total of eleven Chinese experts, including five senior TCM practitioners (three experts major in Chinese herbal medicines and two experts major in acupuncture and moxibustion), three evidence-based medicine and clinical trial methodology experts, two reporting guideline developers, and one epidemiologist, accepted our invitation and attended this meeting. During the meeting, the study background and the suggested items were presented to all participants, followed by a discussion and revision of each item. Finally, a checklist of extension reporting items was formed and the experts also suggested to test the checklist through receiving broad comments and feedbacks.

(2) ***Prepare and conduct a three-round of Delphi survey:***

Delphi exercise was followed to further test and modify the extension items. Firstly, two researchers (JC and LL), experts in Computer Science, developed the online Delphi questionnaires and maintained the stability of the web-based system. The Qualtrics Survey Software (Hong Kong Baptist University) was used in the investigation. Secondly, we invited 50 professionals by email-based invitation which including the objective and introduction of this study, explanations of the Delphi exercise, as well as the informed consent of comments collection and confidentiality of responses during the Delphi process. Thirdly, a total of 42 experts (response rate: 84%) agreed to the participation of the Delphi survey. These individuals represent a broad range of disciplines as well as diverse cultures and geography (Additional file 1). Then, we assigned each participant a unique registration number and sent them out the available link of the online questionnaire with the validity period for submission.

A three-round Delphi survey was conducted from November 2018 to April 2019. Among 42 participants, 71.4% (30/42) completed all rounds of the Delphi, while the remaining 28.6% (12/42) finished two rounds of the questionnaires. During the process, participants were asked to rate each item on a scale of 1 (not important) to 5 (very important), suggest new items, and to provide comments (if any) for each item (e.g., reasons for why they believed the item that should or shouldn’t be extended); any items that did not reach consensus or any new items and comments summary of each item were circulated in subsequent rounds. Following each round, the score for each item was calculated with the formula of 100% * (1*N_5_ + 0.75*N_4_ + 0.5*N_3_ + 0.25*N_2_) / (N_5_ + N_4_ + N_3_ + N_2_ + N_1_), where N_i_ represents the number of respondents who chose specific “i” in the scale of “1 to 5”. Items with a score greater than or equal to 75% were included. The above calculation formula was referred to the RIGHT (A Reporting Tool for Practice Guidelines in Health Care) statement, where both the consensus level and the weight of responses were considered [24]. An anonymized summary of the results of each round was sent to all participants through emails with the questionnaire of the following round. The collection and analysis of the data were administrated by our working group members (XZ, RT and ZB).

(3) ***Finalizing the checklist and Explanation & Examples:***

According to the results of Delphi survey, the inclusion items were determined in the checklist (Additional file 2). In addition, all comments from the Delphi participants were summarized and analyzed by our working group members. Some valuable comments on the rationale of extension items were added or modified in the preparation of explanation and elaboration (E&E) documents. For the examples of good reporting, most of them were extracted from the registered record with a higher quality score in our previously baseline survey. After that, the checklist with an E&E document was presented to the advisory expert group for further revision.

The advisory group was established in June 2019 and composed of seven professionals with international reputations, specifically including two researchers who have profound qualifications in the fields of reporting guideline from Canada, two clinical trial registration experts from Switzerland, one experienced editor from the United Kingdom, and two experts of evidenced-medicine and good clinical practice from Hong Kong. They reviewed and modified the checklist, as well as provided constructive comments and suggestions for the presentation of E&E document. Finally, the working group members have further revised and finalized the manuscript of this WHO TRDS-TCM 2020 statement at the end of 2019.

## Results

### Highlights of WHO TRDS-TCM 2020

The WHO TRDS-TCM 2020 checklist finally includes a total of 11 extension item for TCM, which highlighted the fundamental concepts of TCM Pattern [17] and the features of three TCM interventions, namely CHM formula, acupuncture and moxibustion. Compared with the standard items of TRDS (V.1.3.1), this Extension elaborates on ten of the 24 TRDS items and one of the optional items. The total 11 items are: Source(s) of Monetary or Material Support (Item 4), Scientific Title (Item 10a and 10b), Countries of Recruitment (Item 11), Health Condition(s) or Problem(s) Studied (Item 12), Intervention(s) (Item 13a, 13b and 13c), Key Inclusion and Exclusion Criteria (Item 14), Primary and Key Secondary Outcomes (Item 19 to 20), and Lay Summary (B1). The checklist is presented in the Table 1; elaborations of TCM extensions are italicized. Explanations of corresponding items are given below, and available examples of good registration are provided in Additional file 3.

**Table 1:** Checklist of Items for Clinical Trial Registration of Traditional Chinese Medicine^a^

Number	Original Item/Label ^b^	Explanatory text ^b^	Extension for TCM
1	Primary Registry and Trial Identifying Number		
2	Date of Registration in Primary Registry		
3	Secondary Identifying Numbers	Other identifiers besides the Trial Identifying Number allocated by the Primary Registry, if any. These include: • The Universal Trial Number (UTN) • Identifiers assigned by the sponsor (record Sponsor name and Sponsor-issued trial number (e.g. protocol number)) • Other trial registration numbers issued by other Registries (both Primary and Partner Registries in the WHO Registry Network, and other registries) • Identifiers issued by funding bodies, collaborative research groups, regulatory authorities, ethics committees / institutional review boards, etc. All secondary identifiers will have 2 elements: an identifier for the issuing authority (e.g. NCT, ISRCTN, ACTRN) plus a number. There is no limit to the number of secondary identifiers that can be provided.	
4	Source(s) of Monetary or Material Support	Major source(s) of monetary or material support for the trial (e.g. funding agency, foundation, company, institution).	*Statement of whether any conflicts of interest exist.*
5	Primary Sponsor	The individual, organization, group or other legal entity which takes responsibility for initiating, managing and/or financing a study. The Primary Sponsor is responsible for ensuring that the trial is properly registered. The Primary Sponsor may or may not be the main funder.	
6	Secondary Sponsor(s)	Additional individuals, organizations or other legal persons, if any, that have agreed with the primary sponsor to take on responsibilities of sponsorship. A secondary sponsor may have agreed to: • take on all the responsibilities of sponsorship jointly with the primary sponsor; or • form a group with the Primary Sponsor in which the responsibilities of sponsorship are allocated among the members of the group; or • act as the Primary Sponsor’s legal representative in relation to some or all of the trial sites.	
7	Contact for Public Queries	Email address, telephone number and postal address of the contact who will respond to general queries, including information about current recruitment status.	
8	Contact for Scientific Queries	Responsibility for scientific leadership must be clearly assigned to a named Principal Investigator. The PI may delegate responsibility for dealing with scientific enquiries to a scientific contact for the trial. This scientific contact will be listed in addition to the PI. The contact for scientific queries must therefore include: • Name and title, email address, telephone number, postal address and affiliation of the Principal Investigator, and; • Email address, telephone number, postal address and affiliation of the contact for scientific queries about the trial (if applicable). The details for the scientific contact may be generic (that is, there does not need to be a named individual): e.g. a generic email address for research team members qualified to answer scientific queries.	
9	Public Title	Title intended for the lay public in easily understood language.	
10	Scientific Title	Scientific title of the study as it appears in the protocol submitted for funding and ethical review. Include trial acronym if available.	*10a. Statement of whether the trial targets a TCM Pattern, or a Western medicine–defined disease, or a Western medicine–defined disease with a specific TCM Pattern.* *10b. Illustration of the name of the TCM intervention, in terms of 1) Chinese herbal medicine (CHM) or CHM formula, 2) acupuncture, 3) moxibustion, or 4) other TCM therapies (*i.e. *cupping, Taichi,* etc.*).*
11	Countries of Recruitment	The countries from which participants will be, are intended to be, or have been recruited at the time of registration.	*The research setting(s) or centre(s) from which participants will be, are being, or have been recruited at the time of registration.*
12	Health Condition(s) or Problem(s) Studied	Primary health condition(s) or problem(s) studied (e.g., depression, breast cancer, medication error). If the study is conducted on healthy human volunteers belonging to the target population of the intervention (e.g. preventive or screening interventions), enter the particular health condition(s) or problem(s) being prevented.	*If the study is conducted on participants with a TCM Pattern, or a Western medicine–defined disease with a specific TCM Pattern, enter the specific name(s) of TCM Pattern(s) studied (*e.g.*, qi deficiency pattern, deficiency of stomach yin pattern, qi stagnation pattern).*
13	Intervention(s)	For each arm of the trial record a brief intervention name plus an intervention description. Intervention Name: For drugs use generic name; for other types of interventions provide a brief descriptive name. For investigational new drugs that do not yet have a generic name, a chemical name, company code or serial number may be used on a temporary basis. As soon as the generic name has been established, update the associated registered records accordingly. For non-drug intervention types, provide an intervention name with sufficient detail so that it can be distinguished from other similar interventions. Intervention Description: Must be sufficiently detailed for it to be possible to distinguish between the arms of a study (e.g. comparison of different dosages of drug) and/or among similar interventions (e.g. comparison of multiple implantable cardiac defibrillators). For example, interventions involving drugs may include dosage form, dosage, frequency and duration. If the intervention is one or more drugs, then use the International non-proprietary name for each drug if possible (not brand/trade names). For an unregistered drug, the generic name, chemical name, or company serial number is acceptable. If the intervention consists of several separate treatments, list them all in a series, separated by commas (e.g. “low-fat diet, exercise”). For controlled trials, the identity of the control arm should be clear. The control intervention(s) is/are the interventions against which the study intervention is evaluated (e.g. placebo, no treatment, active control). If an active control is used, be sure to enter in the name(s) of that intervention or enter “placebo” or “no treatment” as applicable. For each intervention, describe other intervention details (dose, duration, mode of administration, etc.).	*13a. Descriptions of TCM interventions.* *Details for the three most common interventions (Chinese herbal medicine formulas, acupuncture and moxibustion) are given below:* • *Chinese herbal medicine formulas* *1) For fixed CHM formulas: name (*e.g.*, Chinese Pinyin, Latin, or English), source (if any), dosage form, dosage and administration route of the CHM formula; name and dosage of each medical substance.* *2) For individualized CHM formulas: add the rationale/criteria for modifying the formula.* *3) For patent proprietary CHM formulas: add a statement of whether the formula used in the trial is for a condition that the formula is originally targeted.* • *Acupuncture* *1) The names (or location if without standard name) of points (uni/bilateral) used, in Chinese (Pinyin) and international code; depth estimation of insertion (if any); the criteria of response sought (*e.g.*, De-qi or muscle twitch response); needle stimulation (*e.g.*, methods of tonifying, or reduction, or even reinforcement and reduction); needle retention time; needle type, if applicable; number of treatment sessions, frequency and duration of treatment sessions.* *2) For electroacupuncture, the planned implementation requirements or criterion (*e.g.*, mode of stimulation (continuous, dense disperse), waveform and stimulus intensity). It is also recommended to provide, the brand and manufacturer of the utilized apparatus.* • *Moxibustion* *The materials used for moxibustion; names (or location if no standard name) of points (uni/bilateral) used for moxibustion, in Chinese (Pinyin) and international code; procedure and technique for moxibustion; criteria for response sought (*e.g.*, warm feeling); number of treatment sessions, frequency and duration of treatment sessions.* *13b. Descriptions of control group(s).* *For interventions with the control group(s), descriptions of the control groups should include the following:* • *For CHM formulas* *1) Placebo control: name and amount of each ingredient (if applicable); description whether the placebo is the physical identical to the tested drug and pharmacological inert (if any); quality control and safety assessment (if any); administration route, regimen, and dosage; production information (*e.g.*, planned manufacturer).* *2) Active control: if a CHM formula was used, see recommendations for CHM formulas above; if a chemical drug was used, the name, administration route, dosage and regime should be reported.* • *For acupuncture or moxibustion* *1) Blank/waitlist control: special arrangement(s) during pre-treatment, treatment and post-treatment periods.* *2) Sham acupuncture or sham moxibustion: details in accordance with the recommendations for acupuncture or moxibustion above. For example, key information of sham acupuncture control should include needling (penetrating or non-penetrating the skin), acupoint (non-acupoint/ irrelevant acupoint), and manipulation (non- or low- grade manipulation).* *13c. Statement of the qualifications or experiences criteria of possible treatment providers, if applicable.*
14	Key Inclusion and Exclusion Criteria	Inclusion and exclusion criteria for participant selection, including age and sex. Other selection criteria may relate to clinical diagnosis and co-morbid conditions; exclusion criteria are often used to ensure patient safety. If the study is conducted on healthy human volunteers not belonging to the target population (e.g. a preliminary safety study), enter “healthy human volunteer”.	*Statement of whether participants with a specific TCM Pattern will be recruited, in terms of 1) diagnostic criteria and 2) inclusion and exclusion criteria, if applicable. All criteria used should be universally recognized, or reference given to where detailed explanation can be found.*
15	Study Type	Study type consists of: 1. Type of study (interventional or observational) 2. Study design including: Method of allocation (randomized/non-randomized) Masking (is masking used and, if so, who is masked) Assignment (single arm, parallel, crossover or factorial) Purpose 3. Phase (if applicable) For randomized trials: the allocation concealment mechanism and sequence generation will be documented.	
16	Date of First Enrollment	Anticipated or actual date of enrolment of the first participant.	
17	Sample Size	Sample Size consists of: 1. Number of participants that the trial plans to enrol in total. 2. Number of participants that the trial has enrolled.	
18	Recruitment Status	Recruitment status of this trial: 1. Pending: participants are not yet being recruited or enrolled at any site 2. Recruiting: participants are currently being recruited and enrolled 3. Suspended: there is a temporary halt in recruitment and enrolment 4. Complete: participants are no longer being recruited or enrolled 5. Other	
19	Primary Outcome(s)	Outcomes are events, variables, or experiences that are measured because it is believed that they may be influenced by the intervention. The Primary Outcome should be the outcome used in sample size calculations, or the main outcome(s) used to determine the effects of the intervention(s). Most trials should have only one primary outcome. For each primary outcome provide: 1. The name of the outcome (do not use abbreviations) 2. The metric or method of measurement used (be as specific as possible) 3. The timepoint(s) of primary interest Outcome Name: Depression Metric/method of measurement: Beck Depression Score Timepoint: 18 weeks following end of treatment	*If TCM-related outcome (*e.g.*, Pattern outcome) involved, illustration of method of measurement in detail, if applicable.*^*c*^
20	Key Secondary Outcomes	Secondary outcomes are outcomes which are of secondary interest or that are measured at timepoints of secondary interest. A secondary outcome may involve the same event, variable, or experience as the primary outcome, but measured at timepoints other than those of primary interest. As for primary outcomes, for each secondary outcome provide: 1. The name of the outcome (do not use abbreviations) 2. The metric or method of measurement used (be as specific as possible) 3. The timepoint(s) of interest	
21	Ethics Review	The ethics review process information of the trial record in the primary register database. It consists of: 1. Status (possible values: Not approved, Approved, Not Available) 2. Date of approval 3. Name and contact details of Ethics committee(s)	
22	Completion date	The date on which the final data for a clinical study were collected (commonly referred to as, “last subject, last visit”).	
23	Summary Results	It consists of: 1. Date of posting of results summaries 2. Date of the first journal publication of results 3. URL hyperlink(s) related to results and publications 4. Baseline Characteristics: Data collected at the beginning of a clinical study for all participants and for each arm or comparison group. These data include demographics, such as age and sex, and study-specific measures. 5. Participant flow: Information to document the progress and numbers of research participants through each stage of a study in a flow diagram or tabular format. 6. Adverse events: An unfavorable change in the health of a participant, including abnormal laboratory findings, and all serious adverse events and deaths that happen during a clinical study or within a certain time period after the study has ended. This change may or may not be caused by the intervention being studied. 7. Outcome measures: A table of data for each primary and secondary outcome measure and their respective measurement of precision (eg a 95% confidence interval) by arm (that is, initial assignment of participants to arms or groups) or comparison group (that is, analysis groups), including the result(s) of scientifically appropriate statistical analyses that were performed on the outcome measure data, if any. 8. URL link to protocol file(s) with version and date 9. Brief summary	
24	IPD sharing statement	Statement regarding the intended sharing of deidentified individual clinical trial participant-level data (IPD). Should indicate whether or not IPD will be shared, what IPD will be shared, when, by what mechanism, with whom and for what types of analyses. It consists of: 1. Plan to share IPD (Yes, No, Undecided) 2. Plan description	
Additional data items required			
A1	URL	The unique URL of the trial record in the primary registry database.	
Optional data items for collection by the registries			
B1	Lay Summary	Short description of the primary purpose and background of the study followed by a description of the included participants, interventions to be tested and outcomes to be measured. Include a brief statement of the study hypothesis. This should be written in language intended to be read and understood by the lay public. Do not include the entire protocol; do not duplicate information recorded in other data elements.	*Provide a brief statement regarding the specific TCM intervention for a TCM Pattern, a Western medicine–defined disease, or a Western medicine–defined disease with a specific TCM Pattern, as well as a short description of relevant rationale and selection principle of the utilized TCM intervention(s) with references.*
B2	Approvals	Oversight entities that have approved the trial (or to which the trial has been submitted for approval). These include ethics committees and regulatory authorities. For each approving entity the name of the entity, the date and status of the approval should be reported.	

^a^ The original items of WHO Trial Registration Data Set (TRDS) (Version 1.3.1) are provided; elaborations for TCM extensions are in italicized text. We strongly recommend reading this checklist in conjunction with the “WHO International Standards for Clinical Trial Registries” [12] for important clarifications on all original items of WHO data set

^b^ From the Reference [12]

^*c*^ This extension item is used for both original items 19 and 20

### Explanations of WHO TRDS-TCM 2020

#### Item 4: source(s) of monetary or material support

##### TCM extension item: statement of whether any conflicts of interest exist

Authors should report the sources of funding for the trial, as this is essential information for readers assessing a trial. An increasing number of TCM clinical trials, especially CHM interventional trials, are funded by the pharmaceutical industry. Studies have shown that research sponsored by the pharmaceutical industry are more likely to produce results favoring the product made by the company sponsoring the research than studies funded by other sources [25]. The level of involvement by a funder and their influence on the design, conduct, analysis, and reporting of a trial varies. It is therefore important that the authors describe in detail the role of the funders [26]. For the records of trial registration, we suggest researchers to report whether any conflicts of interest will possibly exist between the funder and research. And we also want to point out that it is necessary to apply this requirement to all trial registration, not just for TCM.

#### Item 10: scientific title

##### TCM extension items

**10a. Statement of whether the trial targets a TCM Pattern, or a Western medicine–defined disease, or a Western medicine–defined disease with a specific TCM Pattern.**

**10b. Illustration of the name of the TCM intervention, in terms of 1) Chinese herbal medicine (CHM) or CHM formula, 2) acupuncture, 3) moxibustion, or 4) other TCM therapies (i.e. cupping, Taichi, etc.).**

Every trial should have a self-explanatory scientific title, which provides a first description of the trial design and research question. For trial registration, both public title (e.g., Item 9) and scientific title (e.g., Item 10) are needed. While the public title should be written to easily understood by the laymen, the scientific title is necessarily technical; it must include all elements of the PICOS (Participants, Intervention, Comparator, Outcomes and Study design). In general, the scientific title of a trial as it appears in the protocol submitted for funding and ethical review can be used [12]. For TCM trial registrations, the specific name of target disease and/or TCM Pattern and the TCM intervention(s) should be at least reported.

If the TCM pattern(s) will be involved in participant recruitment of a trial, the registered scientific title should provide the standard name(s), such as “spleen-deficiency syndrome”, or a generalized term of “TCM syndrome differentiation” or “TCM pattern-based”, indicating that Pattern(s) will be studied. For TCM interventions, we also recommend researchers to report the specific name (e.g., style of intervention), if applicable. For example, Chinese herbal medicines are typically administered as either single herbs or formulas; names of them can be presented in English, in Chinese Pinyin, or as the acronym [27], such as “Cordyceps sinensis”, “Xijiao Dihuang Decoction” [28], “PSORI-CM01(YXBCM01) Granule” [29], etc. In comparison, the non-herbal therapies include acupuncture, moxibustion, massage, cupping, Taichi, etc., and their names can be specified in terms of materials used and the details of procedures. With moxibustion as an example, the style of moxibustion used could be direct moxibustion, indirect moxibustion, medicinal moxibustion, heat-sensitive moxibustion, warming needle moxibustion, and natural moxibustion [16]. If a trial intends to include a broad category of TCM interventions (e.g., complex/comprehensive interventions), an overall, summary name, such as Chinese medicine antiviral therapy [30], could be used in the title; in such a case mentioning every intervention would result in an unreasonably long title.

#### Item 11: countries of recruitment

##### TCM extension item: the research setting(s) or Centre(s) from which participants will be, are being, or have been recruited at the time of registration

Although reporting the recruitment countries of a study would be sufficient at the time of registration, some registries require reporting the research settings or centres, at least provide the name(s). For example, the Chinese Clinical Trial Registry (ChiCTR) item, “Countries of recruitment and research settings”, requests information regarding Province, City, Institution hospital and Level of the institution as well as country [31]. The Clinical Research Information Service-Republic of Korea (CRiS) item, “Name of Study Site” [32]; International Standard Randomized Controlled Trial Number Register (ISRCTN) item, “Locations”, requests Countries of recruitment and Trial participating centre [33]. The Clinical Trials Registry-India (CTRI) requests “Countries of Recruitment and Sites of Study” [34]; the Iranian Registry of Clinical Trials (IRCT) and Pan African Clinical Trial Registry (PACTR) request “Recruitment centres” [35, 36]. The research setting is a factor in assessing the nature and credibility of the trial will be conducted; also more generally it is used as a part of descriptions for trial design (e.g., single centre or multi-centre). Thus, we recommend researchers to provide information (e.g., name or total number) about the research setting(s) or centre(s), if applicable.

#### Item 12: health condition(s) or problem(s) studied

##### TCM extension item: if the study is conducted on participants with a TCM pattern, or a Western medicine–defined disease with a specific TCM pattern, enter the specific name(s) of TCM pattern(s) studied (e.g., qi deficiency pattern, deficiency of stomach yin pattern, qi stagnation pattern)

In our previous study, among 2955 TCM interventional trials that registered from 2003 to 2017, there are 376 (12.7%) trials that included the TCM pattern [37]**.** In TCM clinical practice, accurate TCM pattern differentiation is vital for the determination of TCM treatment [38]. If a trial will include TCM Pattern(s), or a Western medicine–defined disease with a specific TCM Pattern, researchers should report the name(s) of primary condition(s) and TCM Pattern(s) studied, such as “Leucopenia (deficiency of both qi and blood syndrome)” [39]. Furthermore, we recommend researchers to use the Pattern name(s) according to the international standards of “WHO International Standard Terminologies on Traditional Medicine in the West Pacific Region” [40] or the “International Standard Chinese-English Basic Nomenclature of Chinese Medicine” [41], which were published by World Federation of Chinese Medicine Societies in 2007. Different from Western condition(s)/disease(s), a TCM pattern may have different translations [42–45]. Thus, the use of internationally recognized, standardized terminology is highly recommended.

#### Item 13: intervention(s)

##### TCM extension items

**13a. Descriptions of TCM interventions.**

*Details for the three most common interventions (Chinese herbal medicine formulas, acupuncture and moxibustion) are given below:*

- *Chinese herbal medicine formulas*
  1. *For fixed CHM formulas: name (*e.g.*, Chinese Pinyin, Latin, or English), source (if any), dosage form, dosage and administration route of the CHM formula; name and dosage of each medical substance.*
  2. *For individualized CHM formulas: add the rationale/criteria for modifying the formula.*
  3. *For patent proprietary CHM formulas: add a statement of whether the formula used in the trial is for a condition that the formula is originally targeted.*
- *Acupuncture*
  1. *The names (or location, if without standard name) of points (uni/bilateral) used, in Chinese (Pinyin) and international code; depth estimation of insertion (if any); the criteria of response sought (*e.g.*, De-qi or muscle twitch response); needle stimulation (*e.g.*, methods of tonifying, or reduction, or even reinforcement and reduction); needle retention time; needle type, if applicable; number of treatment sessions, frequency and duration of treatment sessions.*
  2. *For electroacupuncture, the planned implementation requirements or criteria (*e.g.*, mode of stimulation (continuous, dense disperse), waveform and stimulus intensity). It is also recommended to provide, the brand and manufacturer of the utilized apparatus.*
- *Moxibustion*
  *The materials used for moxibustion; names (or location if no standard name) of points (uni/bilateral) used for moxibustion, in Chinese (Pinyin) and international code; procedure and technique for moxibustion; criteria for response sought (*e.g.*, warm feeling); number of treatment sessions, frequency and duration of treatment sessions.*

A detailed description of TCM intervention(s) at the time of trial registration is beneficial to prevent publication bias and unnecessary duplication, as well as to keep data transparent for the public and healthcare professionals. Generally, the three most common interventions are CHM formulas, acupuncture and moxibustion. Therefore, item 13 (Interventions) is extended to include the registration recommendations for common types of CHM formulas (e.g., fixed, individualized, and patent proprietary), acupuncture (e.g., manual acupuncture and electroacupuncture), moxibustion and their controls (e.g., CHM placebo, active and sham acupuncture/moxibustion). During the development process, the checklist items of SPIRIT-TCM Extension 2018 [18], CONSORT-CHM Formulas 2017 [17], STRICTA (Revised STandards for Reporting Interventions in Clinical Trials of Acupuncture) [15] and STRICTOM (The STandards for Reporting Interventions in Clinical Trials Of Moxibustion) were referred, respectively [16]. Researchers can follow the extension items for each type of intervention.

In all cases, we understand there must be a balance between what is practical and what is ideal; nevertheless, striving for the ideal will improve reporting. In particular, for CHM formulas, the checklist does not set the information about processing, production, quality control and safety assessment as required reporting items in the registration, but this does not mean that such information can be ignored. From the preparation of trial registration, efforts should be made to produce data transparent, comprehensive and accurate. Taking quality control of a CHM formula as an example, reporting any pre-designed quantitative and/or qualitative testing method(s), or providing the name(s) of potential CHM manufacturer would be valuable.

**13b. Descriptions of control group(s).**

*For interventions with the control group(s), descriptions of the control groups should include the following:*

- *For CHM formulas*
  1. *Placebo control: name and amount of each ingredient (if applicable); description whether the placebo is the physical identical to the tested drug and pharmacological inert (if any); quality control and safety assessment (if any); administration route, regimen, and dosage; production information (*e.g.*, planned manufacturer).*
  2. *Active control: if a CHM formula was used, see recommendations for CHM formulas above; if a chemical drug was used, the name, administration route, dosage and regime should be reported.*
- *For acupuncture or moxibustion*
  1. *Blank/waitlist control: special arrangement(s) during pre-treatment, treatment and post-treatment periods.*
  2. *Sham acupuncture or Sham moxibustion: details in accordance with the recommendations for acupuncture and moxibustion above. For example, key information of sham acupuncture control should include needling (penetrating or non-penetrating the skin), acupoint (non-acupoint/ irrelevant acupoint), and manipulation (non- or low- grade manipulation).*

A control group is essential in clinical trials attempting to evaluate the efficacy of an intervention. Among these, the placebo design of TCM interventions has been the topic for years, specifically including placebos for CHM formula, sham acupuncture and sham moxibustion [46]. In our previous study, 889 CHM interventional trials were identified in WHO registries from 1999 to 2017, and 40.8% (363) of them included placebo control. Unfortunately, the quality of CHM placebo design and reporting is often poor [47]. Due to the unique color, taste and smell of CHM formulas, creating a quality placebo, that is, physically identical and pharmacologically inert, is quite difficult [48]. Thus, transparently reporting the details about the placebo, as suggested above, are essential for readers to assess the study design and results.

For sham acupuncture and sham moxibustion, a description of these control interventions, especially about the differences from interventions, should be provided. Specifically, information on penetrating or non-penetrating the skin, non-manipulation or low-grade manipulation, and any variation of the acupoint location should be described for the group of sham acupuncture [49]. For sham moxibustion, how the “sham” situation is achieved, e.g., by burning away from the traditional location or by adding insulation below the moxa, should be stated [16]. Particularly, we encourage researchers to describe what the sham acupuncture or moxibustion is intended to control for, such as for point specificity, or for the type and duration of stimulation. In addition, sources that led to the design of sham acupuncture or moxibustion, such as literature or expert opinion, should also be supplied.

**13c. Statement of the qualifications or experiences criteria of possible treatment providers, if applicable.**

The qualification and experience of treatment providers will influence the nature of the TCM intervention given and is therefore a variable that may significantly affect the trial outcome. Clinical doctors are not necessarily the clinical researchers, and not all researchers of TCM trials have the background of TCM knowledge. Therefore, it is necessary to provide the qualifications or experience of treatment providers, thus to make sure the readers understand who design and implement the trials. For trial registration, researchers could provide a list of who will implement the intervention as well as their qualifications. If not applicable, a pre-designed criteria of treatment providers should be provided, such as the requirements of qualification or professional title, years in TCM practice, or any other experience that may be relevant to the trial interventions. Besides, if the intervention requires patients to provide self-treatment, details of any training in advance or any other measures used for standardizing the intervention should be clearly reported.

#### Item 14: key inclusion and exclusion criteria

##### TCM extension item: statement of whether participants with a specific TCM pattern will be recruited, in terms of 1) diagnostic criteria and 2) inclusion and exclusion criteria, if applicable. All criteria used should be universally recognized, or reference given to where detailed explanation can be found

If the Pattern concept is involved in the participant selection, information of how the Pattern is diagnosed and what criteria are used for including and excluding participants should be described. However, in our previous study, we found that among 376 TCM trial registrations that included Pattern(s) for participants, only 27 (7.2%) trials reported the diagnostic criteria for the Pattern studied. Furthermore, 61 (16.2%) trials did not provide the specific name of the Pattern studied [37]. Such vagueness prevents readers from properly interpreting results and reproducing the study. As recommendations in the CONSORT-CHM formulas 2017 [17], citing nationally or internationally recognized Pattern diagnosis criteria are important to TCM clinical trials. We herewith emphasize that the diagnostic criteria of Pattern should be reported at the time of trial registration, which enables researchers to apply these criteria consistently throughout the trial. We suggest researchers to report the international Pattern standard for clustering of symptoms and signs; if necessary, with detailed explanation or reference(s) where explanations can be found.

#### Item 19: primary outcome(s) and item 20: key secondary outcomes

##### TCM extension item: if TCM-related outcome (e.g., pattern outcome) involved, illustration of method of measurement in detail, if applicable

The validity and reliability of trial outcomes are fundamental to interpretation of results; only valid, reliable results can reflect the true efficacy and safety of a given intervention [50]. The primary outcomes usually appear in the sample size calculation (Item 17), and the number of them should be as small as possible; while the remaining outcomes constitute the secondary outcomes. For TCM interventional trials, the commonly used outcomes can be categorized into Western medicine–specific outcomes and TCM-specific, or Pattern-based, outcomes [51]. The Pattern outcomes are more likely to include symptoms and signs assessed by TCM diagnostic methods [52, 53], which can be measured in terms of occurrence (e.g., presence or absence of symptoms or signs), by a rating scale (e.g., score assessment), or an assessment questionnaire (e.g., validated Pattern survey). If a TCM interventional trial included Pattern outcome(s), the information about the name(s), measurement(s), time point(s) should be reported. The methods for measurements are particularly important. In different trials, even the same Pattern outcome could be assessed with various measures according to different rationale; some researchers may develop their own methods for outcome measurements. Thus, we recommend researchers to report the methods of measurement(s) in detail, such as the assessment procedures and supporting references. To improve the quality of assessment, it is better to provide a statement of who will evaluate Pattern outcome(s) (e.g., certified Chinese medicine practitioners or other trained professionals) and if there will be any special arrangements for Pattern outcome(s) evaluation (e.g., patient diary with series of symptoms and signs).

### Optional data items for collection by the registries

#### B1: lay summary

##### TCM extension item: provide a brief statement regarding the specific TCM intervention for a TCM pattern, a Western medicine–defined disease, or a Western medicine–defined disease with a specific TCM pattern, as well as a short description of relevant rationale and selection principle of the utilized TCM intervention(s) with references

This item is an optional data item for registry collection in WHO TRDS (v.1.3.1), which is not included in the required 24 items. Most registries, however, have individually included relevant registration items regarding “lay summary” of the trial. Different registries use different names. For example, ClinicalTrials.gov (registry of the United States) includes two items, “Brief Summary” and “Detailed Description”, which are related to the B1 item (Lay summary). Specifically, this item involves three reporting aspects: 1) study objective/hypothesis; 2) study background/rationale; and 3) description of participants, interventions, comparisons and outcomes (PICO). In our previous review, it was found that, of 2955 TCM interventional trial registrations, 2844 (96.2%) trials reported the study purpose, 1644 (55.6%) trials provided a short description of PICO, and 972 (32.9%) reported the study background/rationale, particularly based on TCM theories [13].

Firstly, the objectives or hypotheses are the questions that the trial is designed to answer [54]. Whether the TCM intervention(s) targets a Western medicine–defined disease, a Pattern, or a Western medicine–defined disease with a specific Pattern should be clarified, and then readers can easily understand which conditions and treatments are targeted in the trial. Secondly, the background and underlying rationale of the research question can help readers to understand the significance of the trial. For a better understanding of TCM trials, it is recommended that the TCM rationale for selecting the TCM intervention(s) for a targeted disease and Pattern, as well as the therapeutic principles about both the Pattern and intervention should be provided, if applicable. To make this part concise, a literature review or relevant references could be cited. Thirdly, a brief description of the PICO must be clearly presented; this will facilitate application of the trial results in the future clinical practice. Regarding the description of TCM interventions and Patterns, easily understood layman’s language is recommended.

## Discussion

It is critical that each clinical trial of TCM should have a complete and transparent reporting of registration. Registration supports good research, enhances the international recognition of TCM modernization, and helps bring TCM into mainstream medicine. In response to observed deficiencies in TCM trial registrations, the WHO TRDS-TCM 2020 working group has developed Recommendations by extending the updated 24 items of TRDS (V.1.3.1) to TCM specifics. It particularly provides the registration items for three common TCM interventions, that is, CHM formulas, acupuncture and moxibustion. According to the unique characteristics of TCM, the WHO TRDS-TCM 2020 checklist includes the key concept of TCM Pattern and elaborates relevant registration items if the Pattern design will be involved. Also, we recognize that researchers who address the clinical question without pattern-related design may not need to focus the pattern-related extension items for their registration. The overall aim of the guideline is to improve the reporting and transparency of TCM clinical trials from the stage of registration. The WHO TRDS-TCM 2020 Items are a practical resource for researchers to understand and prepare the essential information of a TCM trial registration.

The checklist of WHO TRDS-TCM 2020 was developed through extensive consultations with and solicitation of comments from senior TCM practitioners, clinicians in Chinese and Western medicine, experts in reporting guidelines and clinical trials, epidemiologists and journal editors (Additional file 4). An explicit, transparent, and documented process was followed for its development. The Recommendations should be used together with the original TRDS items for different types of TCM clinical trials. Both interventional and observational trials can reference the framework of registration items. While different registries may have specific formats for their registration records, each item in the WHO TRDS-TCM 2020 checklist should be clearly provided or correspond to one or more of the registry’s items because the requirements for clear, transparent reporting—for good science-- are universal.

For better dissemination of this guideline, we will take the following specific steps. Firstly, we will introduce this guideline to complementary and alternative medicine practitioners, researchers, peer reviewers, and journal editors through academic workshops and conferences. Secondly, we will upload the guideline, checklist and E&E documents to our websites, Chinese Clinical Trial Registry (http://www.chictr.org.cn/searchproj.aspx) and the Hong Kong Chinese Medicine Clinical Study Centre (https://cmcs.hkbu.edu.hk/) for free download. As the primary registry authorised by WHO ICTRP, the ChiCTR will promote endorsement of these Recommendations for TCM trial registrations by WHO ICTRP. Thirdly, trialists with TCM play an important role in trial registration, and their needs will actually decide the use of this checklist. We strongly suggest the trialists of TCM to follow the WHO TRDS-TCM 2020, and send the comments, if any, to our working group. The working group welcomes and will collect comments from those in research or clinical practice in order to revise the reporting items. Fourthly, we will periodically reappraise and further modify the checklist through conducting a large-scale user-based survey to collect feedbacks and test the practicality of each item. In addition, the guideline will be updated by adding registration items of more TCM interventions (e.g., cupping, massage) in the future. Based on above steps, we hope that the potential to improve the completeness and transparency of registration reporting for TCM trials, as well as to limit the number of poor-quality registrations, could be fully realized.

## Conclusion

The WHO TRDS-TCM 2020 aims to help researchers improve the reporting and transparency of TCM clinical trial registrations. We also hope these recommendations will support WHO-registries in assessing the registration quality of TCM clinical trials, and will help readers better understand TCM (e.g., CHM formula, acupuncture and moxibustion) trial design.

## Supplementary information

**Additional file 1.** Description of 42 participants in the Delphi survey.

**Additional file 2.** The flow of three-round Delphi survey.

**Additional file 3.** Available registration examples of good reporting of TCM clinical trials.

**Additional file 4.** Contributors to WHO TRDS-TCM 2020.

## Data Availability

All results data of this study are provided in the manuscript and supplementary files. Data of specific experts’ comments are available from the corresponding author on receiving a reasonable request.

## Abbreviations

CTR: Clinical trial registration
TCM: Traditional Chinese medicine
CHM: Chinese herbal medicine
WHO: World Health Organization
TRDS: Trial Registration Data Set
WHO TRDS-TCM 2020: World Health Organization Trial Registration Data Set for TCM Extension 2020
ICTRP: International Clinical Trials Registry Platform
ICD-11: Eleventh revision of the International Statistical Classification of Diseases and Related Health Problems
CONSORT: Consolidated Standards of Reporting Trials
SPIRIT: Standard Protocol Items: Recommendations for Interventional Trials
PICOS: Participant, Intervention, Control, Outcome and Study design
E&E: Explanation and elaboration
WM: Western medicine
RCT: Randomized controlled trial
ChiCTR: Chinese Clinical Trial Registry
CRiS: Clinical Research Information Service-Republic of Korea
ISRCTN: International Standard Randomized Controlled Trial Number Register
CTRI: Clinical Trials Registry-India
IRCT: Iranian Registry of Clinical Trials
PACTR: Pan African Clinical Trial Registry
STRICTA: Revised Standards for Reporting Interventions in Clinical Trials of Acupuncture
STRICTOM: The STardards for Reporting Interventions in Clinical Trials Of Moxibustion
