# Supplementary material for: WHO Trial Registration Data Set (TRDS) extension for traditional Chinese medicine 2020: recommendations, explanation, and elaboration
Source: BMC Med Res Methodol. 2020 Jul 17;20:192. doi: 10.1186/s12874-020-01077-w (PMC7367238; doi:10.1186/s12874-020-01077-w)
Supplement: Supplementary file 1 — Additional file 1. Description of 42 participants in the Delphi survey. [file 12874_2020_1077_MOESM1_ESM.docx]

**Additional file 1: Description of 42 participants in the Delphi survey**

**Table. General characteristics of 42 participants in the Delphi survey**

| Category | Number (n=42, %) |
| --- | --- |
| **Gender** |  |
| Male | 27 (64.3) |
| Female | 15 (35.7) |
| **Working Experience (Years)** |  |
| ≤10 | 13 (31.0) |
| 10-20 | 17 (40.5) |
| 20-30 | 5 (11.9) |
| >30 | 7 (16.7) |
| **Professional field** |  |
| Clinician in Chinese and Western Medicine | 5 (40.1) |
| Clinician in traditional Chinese medicine | 15 (35.7) |
| Methodologist (Clinical trial or reporting guideline) | 19 (45.2) |
| Editor of medical journal | 1 (2.4) |
| Statistician | 1 (2.4) |
| Epidemiologist | 1 (2.4) |
| **Professional title** |  |
| Senior | 11 (26.2) |
| Middle | 15 (35.7) |
| Primary | 9 (21.4) |
| Others | 7 (16.7) |
| **Institution** |  |
| University | 31 (73.8) |
| Hospital | 5 (11.9) |
| Research institute | 4 (9.5) |
| Journal agency | 1 (2.4) |
| Industry | 1 (2.4) |
| **Geographical distribution** |  |
| China Mainland | 25 (59.5) |
| HKSAR, China | 9 (21.4) |
| Australia | 3 (7.1) |
| Korea | 3 (7.1) |
| UK | 1 (2.4) |
| Norway | 1 (2.4) |
