# Supplementary figures and images for: WHO Trial Registration Data Set (TRDS) extension for traditional Chinese medicine 2020: recommendations, explanation, and elaboration
Source: BMC Med Res Methodol. 2020 Jul 17;20:192. doi: 10.1186/s12874-020-01077-w (PMC7367238; doi:10.1186/s12874-020-01077-w)

**Additional file 2: The flow of three-round Delphi survey**


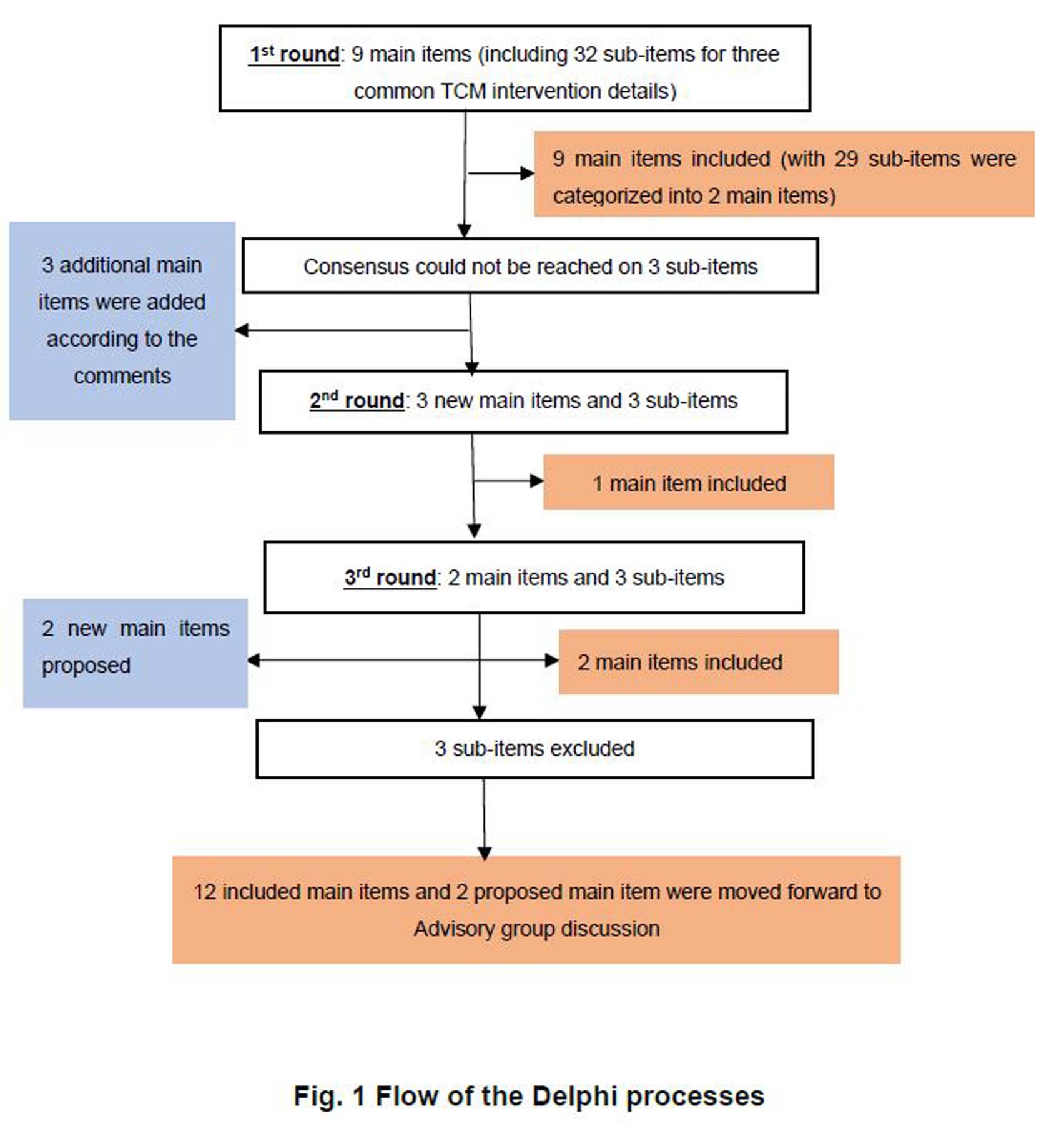

Supplement: Supplementary file 2 — Additional file 2. The flow of three-round Delphi survey. [file 12874_2020_1077_MOESM2_ESM.docx]
