# Supplementary material for: WHO Trial Registration Data Set (TRDS) extension for traditional Chinese medicine 2020: recommendations, explanation, and elaboration
Source: BMC Med Res Methodol. 2020 Jul 17;20:192. doi: 10.1186/s12874-020-01077-w (PMC7367238; doi:10.1186/s12874-020-01077-w)
Supplement: Supplementary file 3 — Additional file 3. Available registration examples of good reporting of TCM clinical trials. [file 12874_2020_1077_MOESM3_ESM.docx]

**Additional file 3: Available registration examples of good reporting of TCM clinical trials**

*Note: We edited the following examples by removing citations or Web addresses for reasons of brevity, and we also spelling out abbreviations for clear understanding.*

**Item 4: Source(s) of Monetary or Material Support.**

TCM Extension item: Statement of whether any conflicts of interest exist.

**Example**

“Conflict of interest: None.”

(There is no available trial registration of TCM including any description of conflict of interest in the item of Source(s) of Monetary or Material Support.)

**Item 10: Scientific Title.**

TCM extension items: 10a. Statement of whether the trial targets a TCM Pattern, or a Western medicine–defined disease, or a Western medicine–defined disease with a specific TCM Pattern.

10b. Illustration of the name of the TCM intervention, in terms of 1) Chinese herbal medicine (CHM) or CHM formula, 2) acupuncture, 3) moxibustion, or 4) other TCM therapies (i.e. cupping, Taichi, etc.).

**Example 1**

“Clinical study of Medicinal Moxibustion with Rhinocort Nasal Spray in the treatment of Allergic rhinitis of spleen-deficiency syndrome” [1].

**Example 2**

“The effect of Yangying Shuxin Dectction on Exercise Capacity in Patients with Heart Failure with Normal Ejection Fraction” [2].

**Item 11: Countries of Recruitment.**

TCM extension item: The research setting(s) or centre(s) from which participants will be, are being, or have been recruited at the time of registration.

**Example**

“Countries of recruitment and research settings:

1. China;

Province：Shanghai;

Institution hospital：Shanghai Municipal Hospital of Traditional Chinese Medicine;

Level of the institution: Tertiary A hospital.

1. China;

Province：Shanghai;

Institution hospital：Shanghai Chest Hospitial;

Level of the institution: Tertiary A hospital.

1. China;

Province：Shanghai;

Institution hospital：Longhua Hospital Shanghai University of Traditional Chinese Medicine;

Level of the institution: Tertiary A hospital.” [3].

**Item 12: Health Condition(s) or Problem(s) Studied.**

TCM extension item: If the study is conducted on participants with a TCM Pattern, or a Western medicine–defined disease with a specific TCM Pattern, enter the specific name(s) of TCM Pattern(s) studied (e.g., qi deficiency pattern, deficiency of stomach yin pattern, qi stagnation pattern).

**Example 1**

“Target disease: deficiency of liver-yin and kidney-yin syndrome” [4].

**Example 2**

“Target disease: Leucopenia (deficiency of both qi and blood syndrome)” [5].

**Item 13: Intervention(s).**

TCM extension item:

13a. Descriptions of TCM interventions.

Details for the three most common interventions (Chinese herbal medicine formulas, acupuncture and moxibustion) are given below:

- Chinese herbal medicine formulas

1) For fixed CHM formulas: name (e.g., Chinese Pinyin, Latin, or English), source (if any), dosage form, dosage and administration route of the CHM formula; name and dosage of each medical substance.

2) For individualized CHM formulas: add the rationale/criteria for modifying the formula.

3) For patent proprietary CHM formulas: add a statement of whether the formula used in the trial is for a condition the formula is originally targeted.

- Acupuncture

1) The names (or location, if without standard name) of points (uni/bilateral) used, in Chinese (Pinyin) and international code; depth estimation of insertion (if any); the criteria of response sought (e.g., De-qi or muscle twitch response); needle stimulation (e.g., methods of tonifying, or reduction, or even reinforcement and reduction); needle retention time; needle type, if applicable; number of treatment sessions, frequency and duration of treatment sessions.

2) For electroacupuncture, the planned implementation requirements or criteria (e.g., mode of stimulation (continuous, dense disperse), waveform and stimulus intensity). It is also recommended to provide, the brand and manufacturer of the utilized apparatus.

- Moxibustion

The materials used for moxibustion; names (or location if no standard name) of points (uni/bilateral) used for moxibustion, in Chinese (Pinyin) and international code; procedure and technique for moxibustion; criteria for response sought (e.g., warm feeling); number of treatment sessions, frequency and duration of treatment sessions.

**13a. Descriptions for three common TCM interventions**

- For fixed CHM formulas, the name, source (if any), dosage form, dosage and administration route.

**Example**

“Intervention: MaZiRenWan (MZRW) Formula.

This CHM used in study is derived from classic text of Chinese medicine (Shang Han Lun, Discussion of Cold-induced Disorders), which can "moisten the intestines, drain heat, promote the movement of qi and unblock the bowel".

MZRW Low dose group: Dissolved MaZiRenWan (MZRW) granule (2.5g/sachet) in 150 ml hot water, take orally twice daily for 8 weeks;

MZRW Median dose group: Dissolved MaZiRenWan (MZRW) granule (5.0g/sachet) in 150 ml hot water, take orally twice daily for 8 weeks;

MZRW High dose group: Dissolved MaZiRenWan (MZRW) granule (7.5g/sachet) in 150 ml hot water, take orally twice daily for 8 weeks.” [6].

- For CHM formulas, the name and dosage of each medical substance.

**Example**

“Chinese herbal medicine formula PROLUNG with individual herbs of the formula are listed as follows (weight in granule preparation): Huang Qi (Radix Astragali Membranaceus): 2 g; Dan Shen (Radix Salviae Miltiorrhizae): 1 g; Jiang Huang (Rhizoma Curcumae Longae): 1 g; Yin Xing (Semen Gingko Bilobae): 1 g; Mai Men Dong (Tuber Ophiopogonis Japonoci): 1 g; Bai He (Bulbus Lilii): 1g; Jin Yin Hua (Flos Lonicerae Japonicae): 1 g; Zi Su Ye (Folium Perillae Frutescentis): 1 g; Yin Yang Huo (Herba Epimedii): 1 g.” [7].

- For individualized CHM formulas, the rationale/criteria for modifying the formula.

**Example**

“During the trial herbal medicines will be administered either as standardised capsules or as individualised herbal granules to be dissolved in hot water to make a herbal drink.

- Standardised CHM treatment will be delivered as herbal capsules via GP practice nurses and will involve the use of fixed herbal formulae for severe episodes and for preventative treatment. These formulae will be developed through expert consultation. They will comprise of three herbs in each formula. Standardised herbs will be administered as 0.4 g capsules (four pills taken twice a day [b.d.] for preventative treatment and four pills taken four times a day [q.d.] in the event of an acute infection).
- Individualised treatment will be administered by experienced practitioners of CHM and will be delivered as concentrated herbal granules that will be dissolved in hot water and drunk. Treatment will be based on the patient-specific diagnosis made by the practitioner and will vary between patients and over time. The individualised arm of the trial will follow the routine practice of CHM, which involves qualified practitioners.” [8].
- For patent proprietary CHM formulas, add a statement of whether the formula used in the trial is for a condition the formula is originally targeted.

**Example**

“Intervention: Ejiao compound (Other Name: Chinese patent medicine): 20ml, twice daily, orally after breakfast; Duration:4 weeks. Ejiao compound is composed with donkey-hide glue, Ginseng, Codonopsis pilosula, prepared rhizome of rehmannia, and crab apple, which has been widely used in the treatment of various types of anemia in China for decades and might be a potentially effective therapy for postpartum anemia. Recently, studies involving animal subjects have helped shed light on its mechanism of action.” [9].

- For acupuncture, The names (or location, if without standard name) of points (uni/bilateral) used, in Chinese (Pinyin) and international code; depth estimation of insertion (if any); the criteria of response sought (e.g., De-qi or muscle twitch response); needle stimulation (e.g., methods of tonifying, or reduction, or even reinforcement and reduction); needle retention time; needle type, if applicable; number of treatment sessions, frequency and duration of treatment sessions.

**Example**

“Intervention: Acupuncture is one of the oldest standardized neuromodulatory therapies. The method of acupuncture treatment involves the insertion of needles in acupuncture points according to a system of channels and meridians. The needles are stimulated by manual manipulation, electrical stimulation or heat. The general theory of acupuncture is based on the premise that there are patterns of energy flow through the body that are essential for health. Disruptions of this flow are believed to be responsible for disease. Acupuncture may correct imbalances of flow at identifiable points close to the skin.

Experimental: Acupuncture: Zhongliao (BL 33), Shenshu (BL 23), Huiyang (BL 35), and Sanyinjiao (SP 6) acupuncture points (Table 1). After patients are in prone position with relax, the investigators will use 75% alcohol pads to sterile the skin around the acupuncture points, and then insert steel needles (Huatuo, Suzhou, China 0.3mm*40mm/0.3mm*75mm) into the acupuncture points. For bilateral Zhongliao (BL 33), the needle will be inserted into about 50-60mm with 45 degree, for Huiyang (BL 35), the needle will be inserted into 50-60mm. for Shenshu (BL 23) and Sanyinjiao (SP 6), the needles will be inserted vertically to a depth of 25-30 mm. The treatment sessions are 24 after baseline, 3 times a week, and the each time the patients will accept a 30 minutes treatment. Acupuncturists will twirl at BL23, BL35 and SP 6 to achieve and enhance the sensation of aches, heaviness or numbness in the area surrounding the inserted needle (known as de qi), and the manipulations will be performed a total of three times during 1 session (every 10 min). For bilateral BL 33, which are located in the 3rd posterior sacral foramina, needles will be inserted without lifting or rotating based on the characteristics of the points.” [10].

- For electroacupuncture, the planned implementation requirements or criteria (e.g., mode of stimulation (continuous, dense disperse), waveform and stimulus intensity). It is also recommended to provide, the brand and manufacturer of the utilized apparatus.

**Example**

“Intervention: Procedure: Electroacupuncture.

The treatment is to be applied twice a week for acupuncture points (unilaterally at CV3, CV4, GV20 and bilaterally at KI3, SP6), used for all the patients assigned to this group. Disposable, sterilized, filiform acupuncture needles manufactured is to be used. An electric acupuncture device (CellMac STN-110, Stratek Co, Republic of Korea) will be connected to the CV3-CV4, KI3-SP6 acupoints and deliver stimulation with a 2-Hz frequency, asymmetric bimodal pulse, continuous wave mode, maximum intensity below the threshold (7.6~13.9mA).” [11].

- For moxibustion, the materials used for moxibustion; names (or location if no standard name) of points (uni/bilateral) used for moxibustion, in Chinese (Pinyin) and international code; number of treatment sessions, frequency and duration of treatment sessions.

**Example**

“Intervention: Drug: Herbal cake, Device: Moxibustion. Herbal cakes formula: Monkshood 10g, Cinnamon 2g, Salvia miltiorrhiza 3g, Flos Carthami 3g, Radix Aucklandiae 2g. Herbs are smashed into powder. Every 2.5g medicine powder is mixed with 3g millet wine and then pressed into herbal cakes using a specific mold. Each cake should be 23mm in diameter and 5mm in height. Then, ignited moxa cones are placed on top of each herbal cake. Herbal cakes with moxa cones will be positioned at acupuncture points ST25, ST37 and CV6. The entire treatment will be done once a day for 1 moxa cone every acupuncture point, 30 minutes at a time, 3 times a week, for a total of 12 weeks.” [12].

- For moxibustion/acupuncture, procedure and technique for moxibustion; criteria for response sought (e.g., warm feeling).

**Example**

“Interventions: Group 1: Experimental acupuncture-moxibustion

Participants in this group receive acupuncture treatment at bilateral acupoints ST37, SP6, SP9, SP4, LR3 and LI3. Sterile disposable stainless-steel needles (with a diameter of 0.30 mm and length of 40 mm or 25 mm) with plastic casings and pedestals are used. The needles are directly inserted 20-30 mm into the skin to elicit a de-qi sensation. The needle is kept in position for 30 minutes. Participants also receive moxibustion treatment at the bilateral acupoints ST25 and ST36. Mild-warm moxibustion is a type of moxa stick moxibustion that is performed by holding an ignited moxa stick a certain distance above the patient's skin, keeping the spot warm and making it reddened, but not burnt. This is done using mild-warm moxibustion that keeps transcutaneous temperature at 43 °C ± 1 °C, for 30 minutes at each acupoint. Moxibustion and acupuncture are performed at the same time once every other day, three times a week, for a total of 24 weeks of treatment.” [13].

TCM extension item:

13b. Descriptions of control group(s).

For interventions with the control group(s), descriptions of the control groups should include the following:

- For CHM formulas

1) Placebo control: name and amount of each ingredient (if applicable); description whether the placebo is the physical identical to the tested drug and pharmacological inert (if any); quality control and safety assessment (if any); administration route, regimen, and dosage; production information (e.g., planned manufacturer).

2) Active control: if a CHM formula was used, see recommendations for CHM formulas above; if a chemical drug was used, the name, administration route, dosage and regime should be reported.

- For acupuncture or moxibustion

1) Blank/waitlist control: special arrangement(s) during pre-treatment, treatment and post-treatment periods.

2) Sham acupuncture or Sham moxibustion: details in accordance with the recommendations for acupuncture and moxibustion above. For example, key information of sham acupuncture control should include needling (penetrating or non-penetrating the skin), acupoint (non-acupoint/ irrelevant acupoint), and manipulation (non- or low- grade manipulation).

**13b. Descriptions of the control group(s)**

- For a placebo of CHM, name and amount of each ingredient; description whether the placebo is the physical identical to the tested drug and pharmacological inert (if any); quality control and safety assessment (if any); administration route, regimen, and dosage; production information (e.g., planned manufacturer).

**Example 1**

“Comparator / control treatment: Placebo.

One standard dose of Chinese herbal medicine placebo, dissolved in warm water and taken orally twice per day for 12 weeks, followed by a wash-out period of 4 weeks and then cross-over to the other arm of the trial. Placebo will be matched to the herbal medicine in color, taste, texture and smell. The placebo consists of dextrin 86%, sucrose 5%, caramel 3.6%, citric acid 1.7%, leaf of broadleaf holly 3.5%, gardenia yellow 300 0.1%, sunset yellow 87 0.05%, lemon yellow 85 0.05% = 100%. The interventions will be manufactured in accordance with Therapeutic Goods Administration (TGA) Australia and Good Manufacturing Process (GMP) guidelines.” [14].

**Example 2**

“Comparator / control treatment: Placebo.

The placebo capsule will be herbal starch that contains no active substances, which will have an identical appearance to the real herbal capsule. The granules of the formula and placebo will be in a standard capsule form produced by a manufacturer that holds TGA approved Good Manufacturing Practice (GMP) certificate. The subject will be asked to take four capsules, three times daily for the period of 12 weeks.” [15].

- For active control, if a chemical drug was used, the name, administration route, dosage and regime should be reported.

**Example**

“Control group: Conventional drug.

Salbutamol Sulphate Inhalation Aerosol (Ventolin®, GlaxoSmithKline Australia Pty Ltd.), 100 μg/press, 200 press, 100 μg each time (when needed), no more than 8 press daily for 12 weeks.

Tiotropium Bromide Powder for Inhalation (Spiriva®, Boehringer Ingelheim International GmbH), 18 μg/capsule, 10 capsule, 18 μg each time, once daily for 12 weeks.

Fluticasone Propionate Powder for Inhalation (Seretide®, Laboratoire GlaxoSmithKline), 50ug/250μg/inhalation, 60 inhalations, 50ug/250μg each time, twice daily for 12 weeks.” [16].

- For blank/waitlist control: special arrangement(s) during pre-treatment, treatment and post-treatment periods.

**Example**

“Waitlist group: The participants in the waitlist group will not receive acupuncture treatment after randomization for 4 weeks, during which period there will be six telephone visits once or twice a week. After this waiting period, they will then receive twice weekly acupuncture sessions for 4 weeks in the same manner as in the acupuncture group. No other interventions during the study period will be allowed and a diary will be collected.” [17].

- For sham acupuncture or sham moxibustion: details in accordance with the recommendations for acupuncture and moxibustion above.

**Example 1**

“Sham comparator: sham acupuncture, sham electroacupuncture, sham moxibustion.

Treatment 2~3 times/week for 8weeks total 20 times. Acupuncture treatment point: Bilateral the arms1: middle for bicepsbrachii muscle belly. Arms 2: upper part 1.5cm by arm1. leg1: upper part 1.5cm by depression at midpoint of the upper border of the paella. leg2: upper part 1/3 of tibia's medial part. leg3: lower part 1.5cm by leg2. Electro stimulation point: leg1. Moxibustion point: bilateral upper part 1.5cm by anterior superior iliac spine at abdomen.

Method: Park sham placebo acupuncture needle using the device performed as a non-invasive. The same accessories of the electroacupuncture equipment will be applied without electric current, but there will be a 'timer' sound and a blinking light. Sham moxibustion is making smoke, not enough thermo stimulation by insulation close the hole that pass thermo stimulation.” [18].

**Example 2**

“Sham bamboo moxibustion: It is composed of 12g salt, 1.2g Artemisia Herb on strainer under the bamboo. There are 2.6g clay on 5mm thickness Styrofoam (the bottom of moxibustion) Average weight of sham moxibustion used experiment is 26.02g. Sham moxibustion is proceeded at cv12 and st36. The middle surface temperature of real moxibustion increased from Yucantan pig’s skin surface (31~32℃) to 49.58±2.159℃. Meanwhile it increased to 38.99±0.712℃ in case of the sham moxibustion. This temperature is under 42℃(effective temperature) so it proves 1) the temperature of sham moxibustion change for blind 2) but under effective temperature. If subjects appeal unbearable fever, moxibustion is moved on both sides(+-3cm) 1-3 times. Subjects fill in questionnaires after each procedure.” [19].

TCM extension item:

13c. Statement of the qualifications or experiences criteria of possible treatment providers, if applicable.

**Example 1**

“Standardised CHM treatment will be delivered as herbal capsules via GP practice nurses…… These formulae will be developed through expert consultation…… Individualised treatment will be administered by experienced practitioners of CHM…... Treatment will be based on the patient-specific diagnosis made by the practitioner and will vary between patients and over time.” [8]

**Example 2**

“Experimental: Electroacupuncture

The patients in this group received electroacupuncture using the same acupuncture points prescribed by a certified Korean Medicine Doctor with more than 6 years of oriental medicine college education and 1 years of clinical experience.

Experimental: Acupuncture

The patients in this group received acupuncture without electric stimulation using the same acupuncture points prescribed by a certified Korean Medicine Doctor with more than 6 years of oriental medicine college education and 1 years of clinical experience.” [11].

**Item 14: Key Inclusion and Exclusion Criteria.**

TCM extension item: Statement of whether participants with a specific TCM Pattern will be recruited, in terms of 1) diagnostic criteria and 2) inclusion and exclusion criteria, if applicable. All criteria used should be universally recognized, or reference given to where detailed explanation can be found.

**Example 1**

“Inclusion Criteria: 1. Participants who have symptomatic knee osteoarthritis, diagnosis of KOA was based on criteria developed by the American College of Rheumatology (ACR) in 1986. 2. The differentiation standard of the arthralgia syndrome of traditional Chinese medicine (TCM): Referring to the Guidelines on the clinical research of Chinese medicine new drugs (2002) ……” [20].

**Example 2**

“Inclusion Criteria: 1. Clinical diagnosis of primary liver cancer (HCC) and colorectal cancer after surgery. 2. Liver cancer and colorectal cancer liver and kidney yin deficiency and spleen qi deficiency syndrome diagnosis standard: Yin deficiency of liver and kidney and spleen qi deficiency syndrome see hypochondriac pain, waist and knee Limp, fever, night sweats, dry mouth and throat, fatigue, anorexia, abdominal distension after eating more, red or pale tongue, less moss or Light peel, pulse fine.” [21].

**Item 19: Primary Outcome(s) and Item 20: Key Secondary Outcomes.**

TCM extension item: If TCM-related outcome (e.g., Pattern outcome) involved, illustration of method of measurement in detail, if applicable.

**Example 1**

“Secondary outcomes: TCM symptoms changes [Time Frame: after intervention at 4 months]. TCM symptoms changes are according to the lung cancer symptom classification quantization table in "Guiding Principles for Clinical Research of Traditional Chinese Medicine in the Treatment of Lung Cancer (2002 Edition)” [22].

**Example 2**

“Secondary outcomes: TCM pattern improvement [Time Frame: 16 weeks]. Clinical remission: clinical symptoms and signs disappear or basically disappear, total scoring declining ≥95%; Excellence: clinical symptoms and signs are significantly improved, total scoring declining ≥ 70%; Effective: clinical symptoms and signs are improved, total scoring declining ≥ 30%; Invalid: clinical symptoms and signs are without obvious improvement or even with exacerbation, total scoring declining < 30%. Remark: calculation formula (nimodipine method) is: [(scoring of prior treatment - scoring of post treatment) / scoring of prior treatment] ×100%.” [23].

**Example 3**

“Primary outcome: Changes in Spleen Qi Deficiency Symptoms Grading and Quantifying Scale (unit: score on a scale) [Time Frame: At baseline and 6, 12, 26, 52 and 104 weeks]. This outcome will be assessed based on the Spleen Qi Deficiency Symptoms Grading and Quantifying Scale (SQD scale) score. Construct of SQD scale: A total of 15 items, of which 7 items assess main symptoms, including stomach pain, stomach distension, acid reflux, abdominal distension, powerless defecation, fatigue and weak, and inappetence; 8 items assess secondary symptoms, including stomach tightness, stomach burn, belching, nausea and vomiting, abnormal stool, tired mind and taciturnity, sallow complexion, and tastelessness and hypodipsia. Scale ranges and explanations: Assessment of each item includes three aspects: degree, frequency in one day, and episodes within one week. The total score of each item is the sum of points of the three aspects. The higher score indicates the worse symptom. The items for assessing main symptoms are double weighted. The score range is 0 to 283 point.” [24].

**Optional data items for collection by the registries**

**B1: Lay Summary.**

TCM extension item: Provide a brief statement regarding the specific TCM intervention for a TCM Pattern, a Western medicine–defined disease, or a Western medicine–defined disease with a specific TCM Pattern, as well as a short description of relevant rationale and selection principle of the utilized TCM intervention(s) with references.

**Example 1**

“Peptic ulcer diseases (PUD) is a common kind of digestive system disease, which mostly involves in stomach and duodenum. PUD may occur at any age but mainly at 30 to 49 years (the proportion was about 50% among all PUD patients). One epidemiological survey showed that the incidence was 17.2% in China and the ratio of gastric to duodenal ulcer diseases was about 1:2.

The two most common reasons for causing PUD were infection of Helicobacter pylori (Hp) and long-term use of non-steroid anti-inflammatory drug (NSAID). In recent year, the infection rate of Hp increased continuously. Among Chinese PUD patients, 92.6% infected Hp, and 72.2% patients who infected Hp occurred PUD. The typical symptoms of PUD were upper abdominal pain, abdominal distension, nausea and vomiting, belching, and inappetence. These symptoms were cyclical, rhythmic and chronic, and were usually tolerable. However, PUD may progress to bleeding or perforation, and these acute complication always lead to worse prognosis.

Along with the development of medical science, traditional Chinese medicine (TCM) is playing an increasingly rule in treatment of PUD. Shen Ling Bai Zhu San is a classic Chinese medicinal formulae originally described in Tai Ping Hui Min He Ji Ju Fang in the Fang Song Dynasty (1102 AD), which is composed of ginseng, tuckahoe, atractylodes, baked licorice, coixenolide, Chinese yam, lotus seed, shrinkage fructus amomi, platycodon grandiflorum, white hyacinth bean, and dried orange peel. It is mainly used for treating the syndrome of spleen qi deficiency, including dyspepsia, chest and stomach distress, borborygmus and diarrhea, limb weakness, thin body, sallow complexion, pale tongue with white and greasy coating, and weak and slow pulse, etc.

In the theory of TCM, spleen is the source for producing qi and blood and thus is the root of life. PUD are classed as the syndrome of "Pi Man (fullness)" in TCM, which was caused by spleen qi deficiency, imbalance of rise and fall, and/or stagnation of qi activity. Shen Ling Bai Zhu San could invigorate spleen by supplying spleen and remove wet, nourish the stomach and intestine, and thus improve symptoms of PUD. Pharmacological researches showed that Shen Ling Bai Zhu San could regulate function of anaerobic and aerobic bacteria in gastrointestinal tract; specifically, it could promote growth of probiotics (e.g. bifidobacterium) and inhibit main drug-resistant strains (e.g. enterococcus), and thereby enhance the body's self-healing function of ulcer.

Jiangzhong Hou Gu® Mi Xi™ is a dietary therapy form of Shen Ling Bai Zhu San, of which removes atractylodes and platycodon grandiflorum (two herbs that could not be used as food) from Shen Ling Bai Zhu San, and adds perilla leaf for adapting a dietary therapy. Jiangzhong Hou Gu® Mi Xi™ used the main formula of Shen Ling Bai Zhu San, so that it could theoretically maintain the treatment effects. Although the reliable health effects of Shen Ling Bai Zhu San has been proved in previous studies, Jiangzhong Hou Gu® Mi Xi™ is optimized in formula and its preparations changed from electuary to rice paste, so that its functional mechanism and efficacy may also be different. Therefore, the investigators plan to perform a hospital-based randomized controlled trial, enroll patients from 13 hospitals in Jiangxi Province in China, aims to assess function and safety of Jiangzhong Hou Gu® Mi Xi™ in patients with spleen qi deficiency and PUD.” [24].

**Example 2**

“Constipation is a common gastrointestinal complaint in clinical practice, which affects in estimated 12%-19% of American, 14% of Asian, and up to 27% of the population depending on demographic factor, sampling, and definition. With the unsatisfactory response to current symptomatic treatments, many patients seek help from traditional Chinese medicine (TCM), mostly by taking Chinese herbal medicine (CHM).

According to the TCM theory, constipation can be broadly divided into two types, excessive and deficient, based on the underlying aetiology. The former is characterized by the presence of Heat or pathological accumulation of Qi. Heat causes constipation by drying the Intestines and the stool. Patients present with hard, dry, pellet like stool, red complexion, dry mouth or halitosis, red tongue with a dry yellow coat, and slippery and rapid pulse. Qi stagnation causes constipation by disrupting the normal movement and descent of Stomach and Intestinal Qi. Patients present difficulty in passing stools, feelings of incomplete evacuation, abdominal distension or pain, frequent belching and flatulence, tongue with thin coat and wiry pulse. The latter, deficient constipation, is delineated as the dryness from insufficient fluid lubrication in the form of blood or lack of propulsion power from the deficiency of Qi or Yang. Besides, the treatment approaches vary from syndrome to syndrome.

The formula of MaZiRenWan (MZRW) composed of six Chinese herbs is firstly recorded in a TCM classic, Discussion of Cold-induced Disorders (Shang Han Lun), and it has been commonly used for constipation in excessive pattern throughout Asia since the Han Dynasty (A.D. 200). By combining the actions of these herbs, MZRW can moisten the Intestines, drain heat, promote the movement of Qi and unblock the bowel. Although previous studies show that MZRW has purgative and laxative effects and may be useful for functional constipation (FC), there are significant methodological weaknesses. Furthermore, the dose of CHM intervention being investigated from the first randomized controlled trial (RCT) published in 1983 is always based on the practitioner's experience, TCM literatures, or experts' comments, but not the results from stringent clinical trials, such as dose determination study. Therefore, the evidence produced will be attenuated or even misleading if improper dose is taken.

In the present study, the efficacy and safety of MZRW in optimal dosage were justified by comparing with placebo under strict clinical trial design.” [25].

**References**

1. ChiCTR-IOR-16008855. Clinical study of Medicinal Moxibustion with Rhinocort Nasal Spray in the treatment of Allergic rhinitis of spleen-deficiency syndrome. URL: <http://www.chictr.org.cn/showproj.aspx?proj=14106>
2. ChiCTR-IOR-17014206. The effect of Yangying Shuxin Dectction on Exercise Capacity in Patients with Heart Failure with Normal Ejection Fraction. URL: <http://www.chictr.org.cn/showproj.aspx?proj=24304>
3. ChiCTR1900022831. Effect of Acupuncture on Lung Cancer-Related Fatigue: A Multi-center Randomized Controlled Trial. URL: <http://www.chictr.org.cn/showproj.aspx?proj=37823>
4. ChiCTR-TRC-14004179. Clinical Trial of 'Gan Shen An' Granules treatment on deficiency of liver-yin and kidney-yin in Type 2 diabetes patients. URL: <http://www.chictr.org.cn/showproj.aspx?proj=5389>
5. ChiCTR-IPR-15006758. Clinical research on Qijiaoshengbai Capsules in treatment of leucopenia (deficiency of both qi and blood syndrome). URL: <http://www.chictr.org.cn/showproj.aspx?proj=11440>
6. NCT00299975. A Dose Determination Study of Chinese Herbal Medicine for Functional Constipation. URL: <https://clinicaltrials.gov/show/NCT00299975>
7. NCT03274544. Clinical Trial of Chinese Herbal Medicine for Idiopathic Pulmonary Fibrosis (IPF). URL: <https://clinicaltrials.gov/ct2/show/record/NCT03274544>
8. ISRCTN95402719. Chinese herbal medicine in the treatment of women with recurrent urinary tract infections. URL: <http://www.isrctn.com/ISRCTN95402719>
9. NCT02656225. Effect of Ejiao Compound in the Treatment of Postpartum Anemia of Qi-blood Deficiency Syndrome. URL: <https://clinicaltrials.gov/ct2/show/NCT02656225>
10. NCT02588274. Efficacy of Acupuncture for Chronic Prostatitis/Chronic Pelvic Pain Syndrome. URL: <https://clinicaltrials.gov/show/NCT02588274>
11. NCT03260907. Efficacy and Safety of Electroacupuncture and Acupuncture in Postmenopausal Women with Overactive Bladder. URL: <https://clinicaltrials.gov/ct2/show/NCT03260907>
12. NCT03232528. The Promotion Plan of Moxibustion on Ulcerate Colitis.

URL: <https://clinicaltrials.gov/ct2/show/NCT03232528>

1. ISRCTN38009074. Acupuncture and moxibustion treatment for Crohn's disease.

URL: <http://www.isrctn.com/ISRCTN38009074>

1. ACTRN12617001247369. Efficacy and safety of a Chinese herbal medicine to reduce hot flushes/night sweats in women treated for breast cancer. URL: <http://www.anzctr.org.au/ACTRN12617001247369.aspx>
2. ACTRN12607000255482. Efficacy and safety of a Chinese herbal medicine formula in the management of simple obesity: Randomised placebo-controlled clinical trial.

URL: <http://www.anzctr.org.au/ACTRN12607000255482.aspx>

1. NCT03169504. Effect of Acupuncture on Patients with Chronic Obstructive Pulmonary Disease.

URL: <https://clinicaltrials.gov/ct2/show/NCT03169504>

1. NCT01921504. Effect of Acupuncture on Patients with Functional Dyspepsia: a Multi-center, Randomized, Waitlist-controlled Trial. URL: <http://clinicaltrials.gov/show/NCT01921504>
2. KCT0001810. Acupuncture and Moxibustion Treatments for the Depression Clinical Trial.

URL: <http://cris.nih.go.kr/cris/en/search/search_result_st01.jsp?seq=5679>

1. KCT0001934. A Sham Moxibustion Device and Masking Test.

URL: <http://cris.nih.go.kr/cris/en/search/search_result_st01.jsp?seq=5709>

1. NCT03309501. Tong-Luo-Qu-Tong Plaster for KOA: a Randomised, Double-blind, Parallel Positive Control, Multi-center Clinical Trial. URL: <https://clinicaltrials.gov/ct2/show/NCT03309501>
2. NCT03189992. Study of TCM Syndrome of Hepatocellular Carcinoma and Colorectal Cancer Based on System Science. URL: <https://clinicaltrials.gov/ct2/show/NCT03189992>
3. NCT03372694. Efficacy Study of Comprehensive Rehabilitation Program Plus Chemotherapy in Postoperative NSCLC Patients. URL: <https://clinicaltrials.gov/ct2/show/NCT03372694>
4. NCT03457324. Efficacy and Safety of Chinese Medicine JCM-16021 for Diarrhea-predominant Irritable Bowel Syndrome. URL: <https://clinicaltrials.gov/ct2/show/NCT03457324>
5. NCT03320538. Efficacy and Safety of Hou Gu Mi Xi in Patients with Peptic Ulcer Diseases. URL: <https://clinicaltrials.gov/ct2/show/NCT03320538>
6. NCT00741936. An Effectiveness and Safety Study of Chinese Herbal Medicine for Functional Constipation. URL: <https://clinicaltrials.gov/ct2/show/NCT00741936>
