# Supplementary material for: WHO Trial Registration Data Set (TRDS) extension for traditional Chinese medicine 2020: recommendations, explanation, and elaboration
Source: BMC Med Res Methodol. 2020 Jul 17;20:192. doi: 10.1186/s12874-020-01077-w (PMC7367238; doi:10.1186/s12874-020-01077-w)
Supplement: Supplementary file 4 — Additional file 4. Contributors to WHO TRDS-TCM 2020. [file 12874_2020_1077_MOESM4_ESM.docx]

**Additional file 4: Contributors to the WHO TRDS-TCM 2020**

**Working group members (in alphabetical order):**

Zhao-xiang Bian (HKSAR, China), Chung Wah Cheng (HKSAR, China), Jacky C.P. Chan (HKSAR, China), Liang Lan (HKSAR, China), Wai Ching Lam (HKSAR, China), Ran Tian (HKSAR, China), Tai-xiang Wu (Sichuan, China), Liang Yao (HKSAR, China), Linda L D Zhong (HKSAR, China), Xuan Zhang (HKSAR, China), and Chen Zhao (HKSAR, China).

**Consensus meeting experts (in alphabetical order):**

Yao-long Chen (Lanzhou, China), You-ping Li (Sichuan, China), Jia Liu (Beijing, China), Jian-ping Liu (Beijing, China), Hong-cai Shang (Beijing, China), Tai-xiang Wu (Sichuan, China), Feng-yun Wang (Shanghai, China), Ke-hu Yang (Lanzhou, China), Chen Yao (Beijing, China), Guo-qing Zheng (Wenzhou, China), and Jun-hua Zhang (Tianjin, China).

**Advisory experts group (in alphabetical order):**

An-wen Chan (Toronto, Canada), Trish Groves (London, UK), Ghassan Karam (Geneva, Switzerland), Ai-ping Lyu (HKSAR, China), David Moher (Ottawa, Canada), Jin-ling Tang (HKSAR, China), and Qi Zhang (Geneva, Switzerland).

**Delphi survey participants (in alphabetical order):**

Terje Alraek (Tromso, Norway), Mark Bovey (London, UK), Yao-long Chen (Lanzhou, China), Ying-Yao Chen (Shanghai, China), Younbyoung Chae (Seoul, Republic of Korea), Xue-jun Cui (Shanghai, China), Hai-yong Chen (HKSAR, China), Hao Chen (Nanjing, China), Ka-kit Tony Chua (HKSAR, China), Chung Wah Cheng (HKSAR, China), Liang Dai (Shanghai China), Shu-fei Fu (Tianjin, China), Tae-Hun Kim (Seoul, Republic of Korea), Joey SW Kwong (HKSAR, China), Myeong Soo Lee (Daejeon, Republic of Korea), Jian-ping Liu (Beijing, China), Ju Ah Lee (Incheon, Republic of Korea), Zhi Liu (Tianjin, China), Jia Liu (Beijing, China), Bo Li (Beijing, China), Xiu-xia Li (Lanzhou, China), Pui Yan Lam (HKSAR, China), Wai Ching Lam (HKSAR, China), Jie-ru Li (HKSAR, China), Shi-lei Ma (Beijing, China), Wen-juan Ma (Lanzhou, China), Hong-cai Shang (Beijing, China), Xin Sun (Sichuan, China), Jin-hui Tian (Lanzhou, China), Tie-jun Tong (HKSAR, China), Gui-hua Tian (Beijing, China), Ze-huai Wen (Guangzhou, China), Feng-yun Wang (Shanghai, China), Ke-hu Yang (Lanzhou, China), Liang Yao (HKSAR, China), Tony Zhang (Victoria, Australia), Christopher Zaslawski (Sydney, Australia), Guo-qing Zheng (Wenzhou, China), Chi Zhang (Beijing, China), Linda L D Zhong (HKSAR, China), Chen Zhao (HKSAR, China), and Ya Zheng (HKSAR, China).
